# Indoor air pollution from secondhand tobacco smoke, solid fuels, and kerosene in homes with active tuberculosis disease in South Africa

**DOI:** 10.1186/s13104-017-2892-2

**Published:** 2017-11-13

**Authors:** Jessica L. Elf, Onyinyechi Eke, Modiehi Rakgokong, Ebrahim Variava, Yudesh Baliram, Katlego Motlhaoleng, Limakatso Lebina, Adrienne E. Shapiro, Patrick N. Breysse, Jonathan E. Golub, Neil Martinson

**Affiliations:** 1 Johns Hopkins Bloomberg School of Public Health, 600 N Wolfe Street, Baltimore, MD 21205 USA; 2 Johns Hopkins School of Medicine, 1550 Orleans Street, Cancer Research Building-2, Baltimore, MD 21231 USA; 3 University of the Witwatersrand, Perinatal HIV Research Unit, SAMRC Soweto Matlosana Collaborating Centre for HIV/AIDS and TB, P.O. Box 114, Diepkloof, 1864, Johannesburg, South Africa; 4 Department of Health North West Province, Department of Internal Medicine, Klerksdorp Tshepong Hospital Complex, Matlosana, 2574 South Africa

**Keywords:** Tobacco, Secondhand smoke, Solid fuel smoke, Kerosene, Air pollution, HIV

## Abstract

**Objectives:**

Secondhand tobacco smoke (SHS), use of solid fuels, and kerosene may play an important role in perpetuating the tuberculosis (TB) epidemic. The purpose of this study was to explore the prevalence of household air pollution (HAP) from these sources in homes of someone with TB in a high HIV-prevalence setting. A convenience sample of homes and household members participating in an ongoing active case-finding study in Matlosana district townships surrounding Klerksdorp, South Africa were included.

**Results:**

We found a high prevalence of air pollution from SHS, solid fuels, and kerosene among individuals in homes with a case of prevalent active TB disease in Klerksdorp, South Africa. Adults in 40% of homes reported a daily smoker in the home, and 70% of homes had detectable air nicotine. In homes with a history of previous TB (prior to but not including the index case) as compared to those without previous TB, both SHS (83% vs. 65%, respectively) and solid/kerosene fuel use for more than 1 h/day (27% vs. 21%, respectively) were more prevalent. Larger studies are needed to estimate the risk of TB from these types of air pollution in HIV infected individuals and settings with high HIV prevalence.

## Introduction

Social and behavioral risk factors are important for tuberculosis (TB) control [1, 2]. Tobacco smoking, for example, is a known risk factor for TB, with an estimated population attributable fraction (PAF) of over 20% for the global burden of disease [1, 3]. Household air pollution (HAP) from burning of biomass fuels and secondhand tobacco smoke (SHS) are known risk factors for other respiratory diseases, however evidence is inconclusive for their association with TB [4–9]. Further, existing research on HAP and TB risk has predominantly been conducted among HIV-uninfected individuals in countries with a low prevalence of HIV. In countries such as South Africa, however, where a large proportion of the burden of TB is due to the concurrent HIV epidemic, research regarding exposure to HAP is limited. HAP in the context of HIV may play an important role in perpetuating the TB epidemic if it increases burden in HIV-uninfected individuals or has a synergistic effect with HIV infection. It is estimated that 18% of South African homes use biomass fuels for cooking, 18% of adults smoke tobacco, and that 29% of youth are exposed to SHS at home [10, 11]. Current evidence suggests that the PAF of TB in South Africa is 6.7% for solid fuel smoke, but does not consider kerosene use or exposure to SHS [2]. This pilot study aims to explore the exposure to HAP from the combustion of wood and kerosene and SHS among individuals in homes of someone with TB in a high HIV-prevalence setting.

## Main text

### Methods

A convenience sample of homes and household members participating in an ongoing active case-finding study for TB in Matlosana district townships surrounding Klerksdorp, South Africa were included in this study [12]. Matlosana is a municipality in the North West Province of South Africa, and consists of the town of Klerksdorp and five residential townships. Clinic support in the study area consists of a public tertiary care hospital and 16 primary care clinics. Matlosana has an antenatal HIV prevalence of close to 30%, a TB incidence rate of 772/100,000 population [13], and unpublished data from previous work suggests over 80% of TB patients are co-infected with HIV.

In the parent study, index TB patients were eligible for inclusion if they were greater than 18 years of age, and were diagnosed with TB by clinical evaluation and radiographic results. Index TB participants must also have initiated TB treatment within the previous 30 days, lived in the district for at least 6 months, reported living with at least one other individual, and consented to a home visit. Index participants were excluded if they did not meet all of these eligibility criteria [12]. All adults (≥ 18 years of age) and children between seven and 17 years of age living in the same household as the index TB case, including the index case themselves, were eligible for inclusion in this air pollution sub-study.

Data collection for the sub-study study took place between July and August of 2012. For each included household, household-level as well as individual-level data was collected. One adult with primary cooking responsibility or knowledge of household fuel use was administered a household survey to collect household-level information, and individual surveys were administered to each eligible and consenting individual in the household. The household survey assessed primary and secondary fuel use, as well as duration of use, for both cooking and heating in the home. The individual questionnaires collected information on tobacco use and exposure to SHS. Individuals were also asked about history of previous TB disease as part of this sub-study, and current prevalent TB disease was ascertained in the parent study by culture confirmation, as previously described [12]. Adults with an unknown HIV status and consenting to a test were provided one as part of the parent study. Adult participants completed their own individual survey, and children’s individual surveys were completed by the child themselves after written consent by the adult primary caregiver and oral assent by the child participant. Passive air nicotine monitors manufactured and analyzed at the Johns Hopkins Bloomberg School of Public Health Secondhand Smoke Exposure Laboratory in Baltimore, Maryland, USA were placed in the common living space of each home for a period of 14 days. A 10% sample of blank monitors and a 10% sample of duplicate monitors were included for quality control purposes.

Descriptive statistics were calculated for variables of interest at the household level. Households were defined as having a TB history if any individual participant in the home reported a past diagnosis of TB (not including the current TB event by the index case). Household-level characteristics of interest were compared across households with and without a TB history using the Chi squared (χ^2^) test, the Fisher’s Exact test, or the Wilcoxon rank-sum test, as appropriate. Households were also dichotomized by detection of air nicotine (non-detectable versus detectable), and household smoking as determined by individual self-report was compared across this dichotomized variable using the Chi squared (χ^2^) test. For all comparisons, statistical significance was defined as p < 0.05. Given this was a pilot study, no formal sample size calculations were conducted.

### Results

In total, 96 adults and 28 children in 53 households were included; 17 of the included adults were the original TB index case for the household, and no other prevalent TB in addition to the index case was found among household contacts in any of the included homes. Of the 60 adults with a known HIV status from previous diagnosis or from testing in the parent study, 25 (42%) were seropositive. In 15 (28%) of the homes, past diagnosis of TB was reported (TB history).

**Fig. 1:**
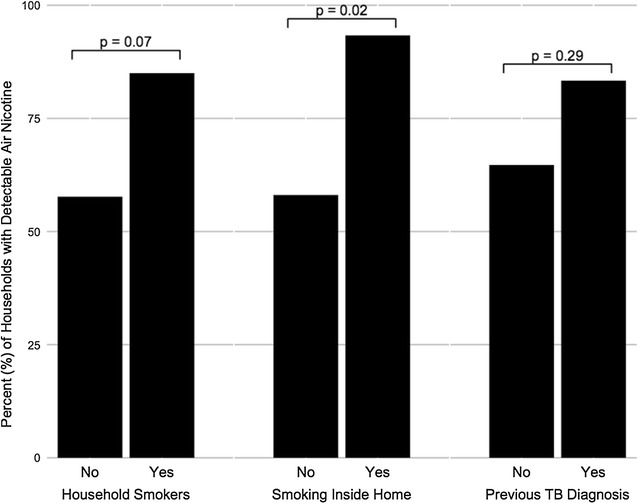
Percent of homes (n = 46) with detectable air nicotine values by reported household exposure

Although electricity was the primary source of energy for cooking in 48 (91%) of homes and one (2%) home used electricity as a secondary fuel for cooking, 9 (17%) homes had a secondary fuel source of either wood or kerosene, 12 (23%) homes used wood or kerosene for cooking, and 8 (15%) of homes used kerosene or wood for heating (Table 1). In total, 16 (30%) of homes reported using either wood or kerosene for cooking or heating. Over one-third (n = 21, 40%) of homes reported a daily smoker in the household. Among homes with air nicotine measurements, however, 32 (70%) had detectable air nicotine (Fig. 1). Further, 14 (58%) of homes where it was reported that no smoking was allowed in the house also had detectable levels of air nicotine. A higher percentage of homes where adults reported a history of past TB diagnosis had detectable air nicotine (n = 10, 83%) than those where no history of TB was reported (n = 22, 65%), although there was not a statistically significant difference (p = 0.29). Similarly, a higher proportion of households with a household history of TB used kerosene or wood for greater than 1 h/day (n = 4, 27%) than those without a household history of TB (n = 8, 21%), though this difference was not statistically significant (p = 0.72).

**Table 1:** Household characteristics of a sample of homes (n = 53) with TB disease in Klerksdorp, South Africa

	Total (n = 53)	Household TB History*	
		No (n = 38, 72%)	Yes (n = 15, 28%)
Housing type, n (%)			
House	43 (81)	28 (74)	15 (100)
Flat	1 (2)	1 (3)	0 (–)
Shack	9 (17)	9 (24)	0 (–)
Number of rooms, median (IQR)	3 (3, 4.3)	4 (3, 5)	3 (3, 4)
Prevalence of household HIV, n (%)	13 (25)	7 (18)	6 (40)
Cooking fuel, n (%)			
Electricity only	41 (79)	30 (79)	11 (73)
Any paraffin/kerosene	10 (19)	6 (16)	4 (27)
Any wood	3 (6)	3 (8)	0 (–)
Any paraffin/kerosene or wood	12 (23)	8 (21)	4 (27)
Secondary cooking fuel source, n (%)	9 (17)	7 (18)	2 (13)
Primary cooking location inside home, n (%)	48 (92)	35 (92)	13 (93)
Use of paraffin/kerosene or wood fuel for heating, n (%)	8 (15)	6 (16)	2 (13)
Any paraffin/kerosene or wood fuel use (cook or heat), n (%)	16 (30)	11 (29)	5 (33)
Cooking with paraffin/kerosene or wood fuel ≥ 1 h/day, n (%)	8 (15)	5 (13)	3 (20)
Heating with paraffin/kerosene or wood fuel ≥ 1 h/day, n (%)	5 (9)	4 (11)	1 (7)
Any paraffin/kerosene or wood fuel use ≥ 1 h/day, n (%)	12 (23)	8 (21)	4 (27)
Daily smoker in household, n (%)	21 (40)	13 (43)	8 (53)
Air nicotine value greater than limit of detection^a^, n (%)	32 (70)	22 (65)	10 (83)

^a^Among homes with an air nicotine monitor (n = 46)

* No statistically significant differences between any of the variables of interest comparing households with and without a history of TB

### Discussion

We report a high prevalence of exposure to air pollution from wood, kerosene, and SHS among individuals in homes with a case of prevalent TB. Nearly one-third of households reported any burning of wood or kerosene for cooking or heating. Moreover, self-report of household SHS exposure was markedly underestimated; the proportion of households with a detectable level of air nicotine was 70%, much higher than the 40% of households reporting a daily smoker in the home. While no prevalent cases of TB in addition to the index case were found, 28% of homes had at least one adult who reported a history of having TB disease in the past. A higher percentage of homes with past TB tended to have a detectable air nicotine, use wood, and use kerosene as compared to homes without past TB.

Given their HIV infection status, people living with HIV (PLWH) are at an increased risk for TB disease. In this high HIV prevalence setting, we found a high prevalence of SHS, which may further increase risk for TB. Globally, 56% of youth report living in homes where they are exposed to SHS, ranging from 42% in the European region to 64% in the Western Pacific. In the African region, 50% of youth reported being exposed to SHS at home [14]. Smoking, and likely subsequent SHS exposure, among people with HIV in South Africa is markedly higher than the general population, where approximately 32% of males and 7% of females smoke [10]. In Klerksdorp, we have previously shown that 52% of men and 13% of women with HIV infection are current smokers [15]. Recent estimates of smoking among TB patients in South Africa have varied, from 56% in active TB patients in Cape Town to 26% in Soweto [16, 17]. We have also shown that among PLWH, smoking triples the odds of TB compared to never smoking [18]. Given the increased risk for TB among smokers, exposure to high levels of SHS as identified in this study, especially in this population, is cause for concern. Further, the present study found a trend for a higher proportion of household smokers and detectable air nicotine in homes reporting a TB history as compared to those with no reported TB history.

Although we found high prevalence of wood for cooking or heating (30%) in our sample, the prevalence of wood used for cooking was lower than previously reported for the South African population in general (18%) [19]. This may be due to the urban location of the households, where greater access to cleaner fuels, less accessible wood, and recent electrification efforts may contribute to different fuel use patterns. This is also much lower than the African region, where in many countries have much lower socioeconomic status as compared to South Africa, and 76–100% of homes use solid fuels. In South-east Asian countries, 51–75% of households use solid fuels, however the burden of HIV is significantly less in this region of the world [19]. Of importance, however, is the relatively high proportion of people reporting using kerosene as a fuel source. Little research has been conducted on the association between kerosene and TB, however evidence suggests exposure to kerosene for cooking and lighting may increase the risk for TB [20, 21]. Given the high prevalence in this vulnerable population, additional research is needed to understand the association between kerosene and TB.

## Limitations

Larger studies are needed to expand upon these findings in high HIV prevalence settings by further categorizing exposure in a larger number of homes and individuals. The small size of the convenience sample included in our study limited our ability to detect statistical differences between households with and without a past history of TB. To accurately estimate prevalence of exposure to HAP in this population, future studies should also employ random sampling among a larger subgroup of the population. As demonstrated by the discrepancy between detectable air nicotine and reported exposure to SHS found in this study, we are also limited in our interpretation of the results as exposure to environmental pollutants are difficult to accurately capture using reported measures alone. In future studies, biological and environmental markers of exposure should be employed. Additional sources of combustion should also be considered, such as the burning of garbage (including plastics and car tires) in the immediate proximity of the home. As exposure to HAP is highly prevalent in this setting, and as the effect of HAP may be different in the context of HIV, more research is needed to understand its contribution to the TB epidemic in settings with high HIV prevalence.

## Abbreviations

HIV: human immunodeficiency virus
IRB: institutional review board
IQR: interquartile range
n: number
%: percent
PAF: population attributable fraction
PLWH: people living with HIV
SHS: secondhand smoke
TB: tuberculosis
χ^2^: Chi squared
